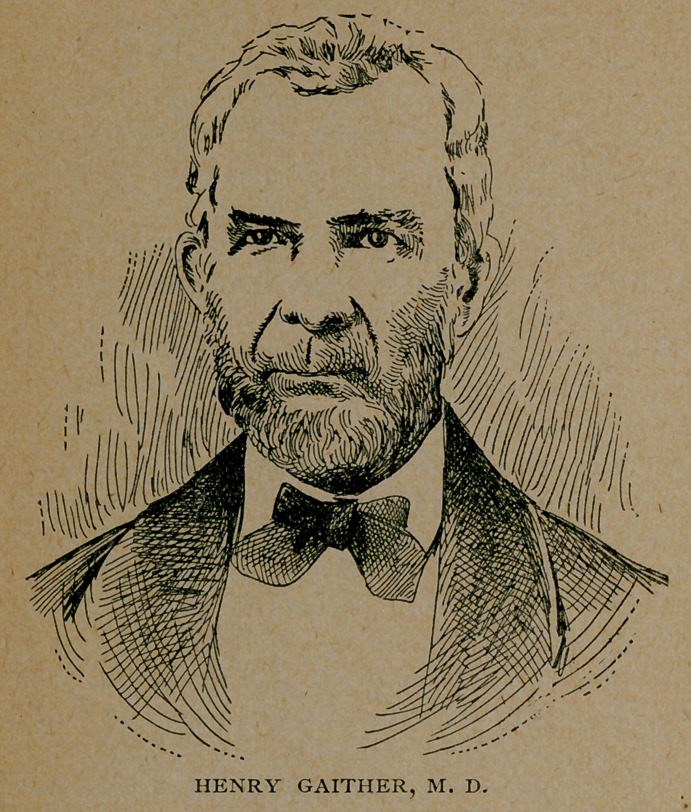# In Memoriam, Henry Gaither, M. D.

**Published:** 1891-10

**Authors:** W. W. Evans

**Affiliations:** Oxford, Ga.


HENRY GAITHER, M. D.

### IN MEMORIAM.

The subject of this sketch was born near Sparta, Hancock county, Georgia, August 8th, 1801. His father was a native of Maryland, and his mother was from Putnam county, Georgia. He received a good country school education, and then an academic training, under the celebrated Alonzo Church, D. D., previous to his elevation to the Presidency of Franklin College, Athens, Georgia. He served in a country doctor's shop; and afterwards went on to New York and became, when quite a youth, a private pupil of the late distinguished and lamented Dr. John W. Francis, of New York City. Subsequently he matriculated in the medical department of the University of Pennsylvania. As a student by competitive examination he became a member of the Medical Society of Philadelphia. After completion of his medical education in Philadelphia, he came to Georgia and began the practice of medicine in Covington in 1825, and after a few year's practice, he took in as junior copartner the late Dr. A. Means, who was the older man, but younger physician. In 1844, he moved to Oxford, Georgia, having been previously elected trustee of Emory College, which place of trust and honor he held as long as he lived. He *never missed a roll call up to the 49th*, that being the last. He joined the Medical Association of Georgia in 1851, two years after its organization; and was an honorary member at the time of his death. The *ad eundem* degree was voluntarily conferred on him by the Medical College of Georgia, at Augusta; it was also done in the same way by the Atlanta Medical College. In April, 1880, the Medical Association of Georgia, at its 31st annual session, held in Augusta, elected him first on the list of delegates to the American Medical Association, at its approaching meeting in New York, in company with Dr. Robert Battey, Henry F. Campbell, J. P. Logan, K. P. Moore and others. He was an *ardent* member of the Medical Association of Georgia, from 1851, the time he joined it, until January 27th, 1891, at which time he passed from among us, surrounded by physicians, family and friends. These two dates—August 8th, 1801, January 27th, 1891, nearly ninety years—embrace a period with him of *no common* living, *for in most* respects he was a very extraordinary man. As Bishop Haygood said of him, "He was brave and heroic, and his sweet spirit, his gentleness, his loving kindness, was the victory of a strong man who, at last, through the grace of God, had triumphed." He possessed property sufficient to have caused many to turn back from the ordinary duties of a village and country doctor, in those days of long rides, by day and by night, over rough roads; and most of the time with no counsel, having to depend entirely upon himself. But he was not of that *stuff*. He loved his profession, and he loved his fellowman and I have often heard him say that "it was as much his love for man, as his love for his profession, that kept him in the trying duties of a country doctor at that time." Fidelity was his motto. Punctuality was *uppermost* in his mind, believing it to be the first element of success in all business. He would be impatient with his best friend, who lacked in this important part of a doctor's make-up. He kept aloof from all office-holding or office-seeking, both civil and military. Refused many times to hold office, keeping an eye single to the study and practice of his profession; and to use his words, "that was sufficient to fill his hands, his head and his heart."

He was a clear, sharp, ready, diagnostician; and thought through a case rapidly, but almost invariably correctly, allowing nothing in the words of the patient or surroundings to keep him from "going to the core of the case," as he expressed it. And after making up his mind, he was firm in his diagnosis and treatment, and would not suffer any deviation in essentials from his plans.

He had no patience with secret nostrums or patent medicines—*was bitter*, and justified himself by saying, if he assumed the responsibility of treatment, he *must know* what he was giving, and would stand by his own prescriptions. He hated charlatanry in all of its forms. He was ever busy in his office, or at the sick bed. He had a contempt for a loafing, gossiping doctor. He had a vein for the humorous, was cheerful and courteous. For pleasure trips and vacations he had but little use, saying, he needed no vacation, he "made a pleasure of business." He was a constant reader of medical journals and also of general literature. He kept up with the profession, not only in reading, but in practice. He had a singular and wonderful gift in selecting from the myriads of new remedies, such as were really useful. At the same time, he would not give up the old things that he knew to be good. Humorously speaking, I used to amuse and at the same time gratify, him, when I said, "I would like to take his picture as an M. D. of sixty-five years of continuous practice with a *thumb lancet*, *fly-blister* and *calomel* in one hand, and a *hypodermic syringe*, *clinical thermometer* and *phenacetine* in the other.

He claimed that the use of stimulants, both in acute and chronic diseases, was rarely necessary; and prided himself that he had "never made a drunkard by the careless use of alcoholic liquors in sickness," saying, "it was better to die a young sober man than an old drunkard." Again, he held that "*no man in health needed stimulants*" to do his work, arguing that the proper use of food and rest, and if necessary, slacking in your work, rather than whip your system up beyond its power, to wear out that much the sooner. Yet he would use stimulants in selected cases. He was wonderfully practical, and used to say that his "mechanical turn had *stood* him good service in treating fractures and other surgical work." Again he was ready to be useful (especially, in the earlier days when such help was more needed than now) in writing wills, or even holding religious services with his patients. I have heard him say he had been "physician, priest and lawyer." For while he was as modest as a woman in his religious pretensions, he was as clear in his faith in the doctrines of christianity as he ever was in any principle in law or medicine. It was not a lowering of his dignity as a doctor to be an honest unpretentious believer. I *never* saw this more clearly exhibited any where than in Oxford, December 29th, 1881, when he had the misfortune to break his right femur from a fall on the back steps of his residence. They were wet and slick from a recent shower of rain, and he had a bucket in one hand and a cup in the other, going to water the flowers in the conservatory. At his age, over eighty, he knew the uncertainties of such an accident, the confinement, etc. But as I entered his room, some little time after, I found him pale as death from the shock, and a clammy sweat on his brow, yet his dark, bright eyes shown with unusual brilliancy, and he had a calm, peaceful, happy expression on his face, and his words abounded in praise to God, for he said, "he accepted it as from him, he did not doubt but that it was in answer to his own prayers, although he had never prayed for his limb to be broken, yet he had asked God to do what was best for him." His wonderful self-possession and readiness in practice were vividly shown in the manner in which he had himself engineered into his room after his fracture. He then called for the yard-stick and took the measurement for the splints himself. Later on he gave directions as to the treatment of his case. Thus exhibiting what one who knew him well had said, that, "while he was faithful and true in all cases of sickness, no matter how common or trivial the complaint, serious diseases and injuries, instead of intimidating him, urged him the more to use the best of his faculties, and as the case progressed he rose with it."

He wrote occasionally for THE ATLANTA MEDICAL AND SURGICAL JOURNAL and other periodicals, and always short, clear and decided articles. No one was ever in doubt as to what he meant, when he wrote or spoke. While his opportunities for early education were meager, he was an educated man, for he had, as Bishop Haygood wrote of him in his obituary, "a ready and masterful use of his faculties." The Bishop also says, "Few men have I known whose fine senses were better trained to quick and accurate observation; he really saw with his eyes and heard with his ears. If his active habits had not been broken up by the breaking of his thigh nine years ago, that vigorous, closely knit frame would, I believe, have done service for more than a hundred years." He did not look or seem to be near as old as he really was. He was in the habit of saying that "he was just as young in his *sympathies*, *sensibilities* and *affections* as he ever was in his life."

He was married twice, first to Miss Sarah Cole, of Greene county, in 1827, and afterwards to Miss Julia A. Fraser, of Marietta, Georgia, in 1871. His success as a physician, surgeon and obstetrician was remarkable—always taking punctually, cheerfully and confidently every case of suffering humanity.

While he honored the great specialists in medicine, he was not ashamed of his place in the rank and file of a general practitioner; holding that while the former deserved praise for consummate skill, the latter deserved none the less for his success in all its branches. The poor he always loved to administer to. He often repeated with *emphasis*, as expressing his views, that notable saying of the late lamented Prof. Joseph A. Eve, M. D., LL. D., of Augusta, Georgia, who said to a graduating class, "*I always want some poor patients*." He used to say, "The lowest bows I make are to the unpretentious in humble life, and to the good in all the walks of life. But for the man who thinks more highly of himself than he ought to think, I have *no special conges to make*."

For nine years after his fracture he was more or less confined to his house, but his uncomplaining spirit, cheerfulness of disposition, and interest in all things was marvelous; and he kept his faithful and devoted wife lovingly busy in reading to him the medical and miscellaneous journals of the day. Physicians sought his counsel in grave cases to the last, and he thus gratified his own feelings and helped others, in doing as he said he intended to do, "*practice to the last*."

If the above sketch should appear to some lengthy and overdrawn, the writer pleads that for its length he hoped by its contents to encourage the younger members of the profession in giving them the secrets of success in "a rare old man who had triumphed. And as to the truthfulness of what I have written a daily medical copartnership of twenty years ought to justify me in knowing and so speaking.

W. W. EVANS, M. D.

*Oxford, Ga., Sept.* 3, 1891.

---

Every week the *Scientific American* presents whatever is new in the world of science, art and manufactures, thus doing service both to theoretical and practical workers. For forty-five years, Munn & Co., 361 Broadway, N. Y., have conducted this paper with close reference to the work of procuring and describing patents in a way to make it an authority on scientific and mechanical subjects.

## Figures and Tables

**Figure f1:**